# Prospects of bacteriophage collections in disinfectant applications

**DOI:** 10.14202/vetworld.2022.220-231

**Published:** 2022-01-31

**Authors:** Samat Serikovich Issabekov, Nazym Syrymkyzy Syrym, Aidar Adilkhanovich Sambetbayev, Kuantar Daulenovich Alikhanov, Bolat Amanbaevich Yespembetov

**Affiliations:** 1 Department of Veterinary Sanitary Examination and Hygiene, Kazakh National Agrarian Research University, Almaty, Republic of Kazakhstan; 2 Laboratory of Microbiology, Research Institute for Biological Safety Problems, Gvardeiskiy Urban-type village, Kordaiskiy region, Zhambylskaya Oblast, Republic of Kazakhstan; 3 Department of Clinical Veterinary Medicine, Kazakh National Agrarian Research University, Almaty, Republic of Kazakhstan.

**Keywords:** bacteriophages, biological preparation, disinfection, lytic activity, strain, titer

## Abstract

**Background and Aim:**

The search and development of disinfectants is promising worldwide. However, there are currently no international regulations governing the testing and registration of germicidal agents. Moreover, the number of safety requirements for disinfectants for human, animal, and environmental health has increased. This research aimed to evaluate the prospects of using a collection of bacteriophages for disinfectant purposes.

**Materials and Methods:**

The objects of research were bacteriophages isolated from a total of 129 environmental samples obtained from seven sources in and around livestock buildings: (1) Feed residues from feeders and automatic drinkers; (2) washouts from floors, walls, and posts; (3) soil from underneath floors; (4) bedding; (5) sewage; (6) ponds; and (7) soil from paddocks. The corresponding strains were used as indicator test cultures for bacteriophages. The authors employed the following methods to work with bacteriophages: (a) Bacteriophage isolation methods, (b) the Appelman method (i.e., serial dilutions), (c) the Grazia method (i.e., agar layers), (d) phage titration on solid media, and (e) the bacterial phagotyping method.

**Results:**

The results of the analysis on the bacteria of the *Enterobacteriaceae* family isolated 11 bacteriophages; one bacteriophage is specific to *Pseudomonas aeruginosa*, and another one is specific to *Brucella abortus*. The results also indicate that all bacteriophage strains of the *Enterobacteriaceae* family demonstrate lysis at a pH of 7.0. In addition, this polyphage lyses all strains of sensitive bacterial cultures. The optimum temperature for the cultivation of bacteriophages is 35°C. While using electron microscopy to study the consortium of bacteriophages, clearly distinguishable virions of bacteriophages were found in the microscope field of view.

**Conclusion:**

The main parameters for the production of polyphages include the ratio of the bacteriophage and its corresponding bacteriophage-sensitive culture, the pH of the cultivation medium, and the cultivation time of the bacteriophage system as well as the sensitive bacterium. With regard to the aforementioned parameters, the results indicate that the average value for all bacteriophages is 1:2, and the average cultivation medium pH is 7.0 for all bacteriophages. The average cultivation time for all bacteriophages is 18-24 h.

## Introduction

The long-term use of antibiotics to treat various diseases has led to the emergence of multidrug resistance in bacterial strains [1-3]. Factors such as the high prevalence of bacterial diseases in Kazakhstan, high annual infection rates among the Kazakh population, and the purchase of animals from other countries require an improvement in the effectiveness of preventive measures to stop the spread of pathogens. The main causative agents of infections circulating in livestock farms are the following genera of bacteria: *Brucella* spp., *Escherichia* spp., *Enterococcus* spp., *Proteus* spp., *Salmonella* spp., *Shigella* spp., *Pseudomonas aeruginosa*, and *Yersinia* spp. The representatives of these families are widespread; they can be isolated from water, soil, and human and animal feces. There is a significant amount of experimental data on the pathogenicity of these microorganisms for animals. *Enterobacteriaceae* can be isolated in cases of mastitis, endometritis, wound infections, and other inflammatory processes in various animal species. The non-fermenting, Gram-negative bacterium *P. aeruginosa*, which can cause various complications in immunocompromised animals, stands apart [4-7]. Given the significant prevalence and circulation of *Pseudomonas aeru­ginosa* in environment, much attention is devoted to the detection of these microorganisms in food products. The search for environmentally friendly ways to decontaminate food products using various phages will increase their shelf life and reduce spoilage.

Conventionally, pasteurization and high-pressure processing methods improve food safety, but they may not be applied to all products; for example, such methods are unsuitable for meat, vegetables, fruits, and berries. Radiation treatment also poses a concern for consumers as it can change the organoleptic properties of food. Chemical disinfection of food is also becoming less popular because chemical disinfectants kill beneficial microflora of the digestive tract, kill pathogenic bacteria and harm the environment. Furthermore, outbreaks of foodborne infections regularly occur even when all the aforementioned methods are employed [8-10].

In recent years, bacteriophages have been considered a promising method for disinfecting food products. This method is called phage biocontrol and is a natural “green” technology that helps target pathogenic microorganisms without affecting beneficial ones. Moreover, bacteriophages are naturally occurring agents that are safe for the environment. Today, all phages that make up the products for biocontrol are wild; that is, after being isolated from natural reservoirs, they have not been subjected to genetic modification. Most phage preparations do not contain chemicals, only an aqueous solution of phages and a low concentration of salts. According to the test data, bacteriophages do not change the organoleptic properties of food products. Finally, the cost of using phage preparations for biocontrol in the food industry is relatively low [11-13].

In the USA and Europe, several bacteriophage preparations for use in the food industry have already been approved by regulatory authorities. These preparations are active against various strains of *Escherichia coli, Listeria monocytogenes, Salmonella* spp., and *Shigella* spp. Some other preparations are being tested or under development [14-16]. Bacteriophages are viruses that selectively attack bacterial cells. The introduction of the phage genome causes the antibacterial effect of bacteriophage preparations into a bacterial cell followed by phage reproduction and lysis of the infected cell. The bacteriophages released in the external environment due to lysis, subsequently reinfect and lyse other bacterial cells until the destruction of pathogenic bacteria in the focus of inflammation [17,18].

Bacteriophages are unique microorganisms that form the basis for a group of therapeutic and prophylactic preparations with unique properties and characteristics. The natural physiological mechanisms of interaction between phages and bacteria that define their action allow for the prediction of an infinite variety of both the makeup of the bacteriophages themselves and their possible applications. With the expansion of bacteriophage collections, new target pathogens will undoubtedly appear, and the spectrum of diseases in which phages can be used both in monotherapy and as part of complex treatment and prevention regimens will also expand.

The modern view on the future of phage therapy should be based on the high specificity of their phage activities and the need to strictly adhere to all the regulations of phage therapy. For biological disinfection, preparations of therapeutic and prophylactic bacteriophages are used. Such preparations contain complexes of polyclonal and virulent (i.e., strictly lytic) bacterial viruses that cause the death of homologous bacterial species due to intracellular reproduction and bacterial cell destruction, accompanied by the release of mature phage particles capable of infecting new bacterial cells [19-21].

Disinfectants used for pathogen decontamination of objects must meet the basic requirements: They must (1) have a pronounced biocidal effect, (2) act within a short microorganism exposure duration, (3) be non-corrosive to metals nor damage other materials present in the objects to be treated, (4) remain active in the presence of organic substances, (5) be convenient to use (e.g., dissolve well, have a long shelf life, and be environmentally friendly), (6) be non-toxic and non-allergenic to humans, and (7) be cost-effective in sum with the equipment used for disinfection [14,16,22,23].

The search and development of disinfectants is a promising field worldwide. However, there are currently no international regulations that govern the testing and registration of germicidal agents. Furthermore, the number of safety requirements has increased for disinfectants to preserve human, animal, and environmental health. Diseases of bacterial etiology pose significant harm to humans and animals and many foods are susceptible to bacterial spoilage. As scientific data on the resistance of bacteria to antibiotics and antimicrobial preparations expand, the use of selective antibacterial preparations, including products that contain bacteriophage complexes, becomes increasingly relevant.

According to various estimates, the world market for bacteriophage preparations is underdeveloped but promising. Bacteriophages can selectively attack a specific bacterial species of various objects. Thus, our patent research into the compositions of bacteriophage products for various purposes demonstrates their usefulness for consumers in numerous countries (e.g., Kazakhstan, Russia, the USA, China, Japan, Australia, Germany, Sweden, Israel, and the Netherlands) and in a wide range of fields (including medicine, veterinary medicine, and everyday applications) [14,22].

The process of creating effective and safe prophylactic phage preparations aims to determine the DNA sequence of promising bacteriophages. After obtaining highly purified preparations of bacteriophages, it is important to detect the possible presence of virulence factors transferred from the bacterial genome to obtain bacteriophages that provide high titers in bacterial infection and thus develop a cost-effective technology.

Analysis of information sources revealed that in the global market, the production of bacteriophage-containing products for indoor cleaning applications is promising and even necessary to reduce the risk of foodborne infections. The scientific work is based on a new innovative technology developed by our team for growing and using bacteriophages in veterinary medicine. The main aim of the technological process is the production of a single biological substance, which consists of a group of bacteriophages, for the subsequent serial production of viable products.

This work aimed to study the prospects of using a collection of bacteriophages for disinfectant purposes.

## Materials and Methods

### Ethical approval

Ethical approval was not necessary for this study as no animals were used in this experiment.

### Study period and location

The research was conducted from February 1, 2018, to December 31, 2019. The experimental part of the research was carried out in the Laboratory of Microbiology at the Research Institute for Biological Safety Problems. The study was carried out using the normative documents officially regulated by the veterinary legislation of the Republic of Kazakhstan. Microbiological samples were collected according to standardized methods.

### Equipment and devices

• A biosafety cabinet, level 2 As24E1, ESCO (Singapore), 2013;
• Electronic laboratory scales SPS 602 F Ekoniks, Professional Equipment and Technologies LLC (PROTEH) Russia, 2014;
• Vacuum pump Laboport N810 FT.18, KNF Neuberger. Supplier: NPP Ekoniks, ERSTVAK LLC, Russia 2014.
• 2014**;** Microscope Mikmed-6var.7SD, Vladmedpostavka, Russia, 2014;
• Refrigerator Polair DM 107-S, VETER LLC, Russia, 2018;
• MSH-300, Magnetic stirrer with a hot plate, cat.BiosanS-010302-OAA, Latvia, 2015;
• Distilling apparatus: ADE-25SZMO, Medoborudovanie JSC 2014, Russia;
• Laboratory centrifuge LMC-4200R, Biosan, 2017, Latvia;
• Sterilization drying cabinet, volume 80 l, ShS-80-01 Smolenskoe SKTB SPU JSC 2018, Russia;
• Sterilizer (Autoclave) Biobase BKHS-360, ACA Group LLP 2017;
• Spectrophotometer Boeco S-30 (Germany), 2013.

### Sampling and data collection

The objects of the study were bacteriophages isolated from environmental samples; a total of 129 samples were examined. The corresponding strains were used as indicator test cultures for bacteriophages. The isolated phages include *Escherichia coli* No. 1, *Proteus vulgaris* No. 13, *Proteus mirabilis* No. 45, *Yersinia pseudotuberculosis* No. 2, *Yersinia enterocolitica* No. 54, *Salmonella enteritidis* No. 15, *Salmonella typhimurium* No. 19, *Salmonella infantis* No. 21, *Enterococcus faecalis* No. 7, *Pseudomonas aeruginosa* No. 8, *Shigella sonne* No. 61, *Shigella flexneri* No. 62, and *B. abortus* No. 57.

### Methods for isolating bacteriophages

Samples of soil and manure weighing 100 g were obtained from objects in the external environment, thoroughly ground in sterile porcelain mortars, and transferred into flasks containing 150 mL of GRM broth. In addition, 120 mL of wastewater samples and surface wipe samples from environmental objects were introduced into flasks containing 30 mL (i.e., concentrated by 5 times) of the same media.

The flasks were placed in a thermostat to incubate for 3 days at 37°C. The incubated material was enriched weekly with 1 cm^3^ thick suspension of *Streptococcus equi* test cultures grown in GRM broth. The flasks were shaken daily to improve the aeration of the incubated mixture. At the end of the incubation period, the mechanical impurities were removed through filtration using a cotton gauze filter; the supernatant was poured into centrifuge tubes and centrifuged at 2500 rpm for 20 min and then filtered using the “Sterile Filtration System” sterilizing filters (with a pore size of 0.2 μm cellulose nitrate and capacity of 115 mL). The filtrate thus obtained was examined for the presence of streptophages.

The activity and specificity of bacteriophages were established on liquid and solid nutrient media through titration with the appropriate type of bacteria using the Appelman and Grazia methods [22].

### The Appelman method (serial dilutions)

In the Appelman method, the determination of bacteriophage activity was performed on the liquid nutrient media by setting the maximum dilution at which complete lysis of the corresponding broth culture of bacteria occurs. This maximum dilution level expresses the titer of a bacteriophage; for example, the titer of a bacteriophage must be at least 10^−7^. The determination of virulence on a solid nutrient medium using the Otto method (i.e., the drop method) consists of the introduction of a drop of a certain dilution of the studied bacteriophage onto the plating of the corresponding culture followed by thermostat processing. The specificity of Brucella bacteriophages is established using the classical method [14].

### The Grazia method (agar layers)

In the Grazia method, the experiment was conducted using the following process: (a) Nutrient agar was poured into Petri dishes and dried in a thermostat, and (b) a semiliquid (0.7%) nutrient agar was prepared, of which 3-4 mL was poured into the test tubes and melted in a water bath. Ten-fold dilutions of the studied phage (10^−2^-10^−7^, depending on the expected titer) were made in an isotonic solution of sodium chloride. Finally, 0.5 mL of the last phage dilution (10^−7^) was mixed with the same volume of a daily broth culture of sensitive-to-phage bacteria and poured into a test tube with semiliquid agar cooled to 45°C. The mixture was quickly poured onto the surface of the agar in a Petri dish, where it solidified into a thin layer.

### Resistance of bacteriophages to the effects of chloroform

We know from the published literature that most bacteriophages are resistant to chloroform. Therefore, this chemical agent is a reliable tool for freeing phagolysate from viable microorganisms. The determination of the sensitivity of bacteriophages to this chemical is performed by processing the phages in a 1:10 ratio with constant stirring followed by the control of the phage titer according to the Grazia method.

### Determination of viral particles

A spectrophotometric method was employed to determine the number of viral particles. Based on the ratio of 2×10^14^ phage particles/mL corresponding to 30 optical units [24], the following formula was used: (A_269_-A_320_) × 10^14^/15, where A_320_ denotes the optical density of the phage suspension at a wavelength of electromagnetic radiation of 320 nm and A_269_ is the optical density of the suspension at a wavelength of 169 nm.

### Determination of virulence

The determination of virulence on a solid nutrient medium using the Otto method (i.e., the drop method) consists of the introduction of a drop of the studied bacteriophage of a certain dilution onto the plating of the corresponding culture followed by thermostat processing. The specificity of *Brucella* bacteriophages is established using the classical method [14].

## Results

In the first test, wastewater was used as the material for bacteriophage isolation. The isolation of bacteriophages was specific to the bacteria of the intestinal group. The control was an indicator culture of the reference strain for each species of the studied bacteria of the *Enterobacteriaceae* family inoculated using the Grazia method of agar layers with 1 mL of sterile meat infusion broth (MIB). The results were recorded after 6-18 h of incubation at 37°C. The presence of bacteriophage was determined by the presence of transparent spots.

One negative colony, located at a distance of at least 10 mm from the other colonies, was selected with a bacteriological loop and inoculated in MIB with an indicator culture specific for each phage species. Simultaneously, the control was set as MIB seeded with an indicator culture without phages. The cultures were incubated at 37°C for 6 h. During this period, the nutrient medium cleared up in the experimental variant and remained turbid in the control variant. The test tube contents were treated with chloroform, and the resulting phagolysate was again examined using the Grazia method of agar layers, in which a negative colony identical to the original one was selected, and the same operation was performed. Using this technique, the isolated phage clones were passaged 7-10 times for each type of bacteria until a phage population with homogeneous negative colonies was obtained. The objects of bacteriophage isolation are presented in Table-1.

**Table-1:** Objects of bacteriophage isolation.

Item No.	Name of the selected phages	Selection source
1	*Echerichia coli* No. 1	Effluents of the dairy farm in the Almaty region
2	*Proteus vulgaris* No. 13	Meat from the Taraz market
3	*Proteus mirabilis* No. 45	Pig farm effluents in Almaty region
4	*Yersinia pseudotuberculosis* No. 2	Cattle breeding farm effluents in the Zhambyl region
5	*Yersinia enterocolitica* No. 54	Shu River in the Zhambyl region
6	*Salmonella enteretidis* No. 15	Poultry farm effluents in the Almaty region
7	*Salmonella typhimurium* No. 19	Poultry farm effluents in the Almaty region
8	*Salmonella infantis* No. 21	Poultry farm effluents in the Almaty region
9	*Enterococcus faecalis* No. 7	Effluents of the Pchelka collective farm in the Almaty region
10	*Shigella sonnei* No. 61	Sorbulak storage tank, Ili District, Almaty
11	*Shigella flexneri* No. 62	Uzun-Kargaly rivers, Ili District of Almaty
12	*Pseudomonas aeruginosa* No. 8	Sewage effluents from a hospital in the Almaty region
13	*Brucella abortus* No. 57	The slurry of the Dostyk farm in the Ili district of the Almaty region

A total of 11 bacteria were isolated as a result of this test. The isolation of bacteriophages was specific to Gram-negative, non-fermenting bacteria of the *Pseudomonas* genus. The test material (10 mL) was inoculated into a flask containing 50 mL of MIB, and broth cultures (1 mL) of each type of bacteria of the *Pseudomonas* genus were added daily. The cultures were incubated at 30°C for 72 h. After incubation, the flasks were stirred by rotation, and 10 mL of the liquid was transferred into sterile test tubes. Next, the phagolysates were examined for the presence of phages. The lytic activity of phages was increased through passivation on indicator cultures of specific bacteria with periodic resowing of negative colonies typical for this phage isolate.

One negative colony, located at a distance of at least 10 mm from other colonies, was selected with a bacteriological loop and inoculated in MIB with an indicator culture specific for each phage species. Simultaneously, the control was set as MIB seeded with an indicator culture without phages. In contrast to the first test, the cultures were incubated at 30°C for 6 h. During this period, the nutrient medium cleared up in the experimental variant and remained turbid in the control variant. The contents of the test tube were treated with chloroform, and the resulting phagolysate was examined again using the Grazia method, selecting a negative colony identical to the original one, with which the same operation was performed. The isolated phage clones were passaged 7-10 times using this technique for each species of *Pseudomonas* bacteria until a phage population with homogeneous negative colonies was obtained.

As a result of the second test, one bacteriophage specific to *P. aeruginosa* was isolated. Isolation of the bacteriophage was specific to *B. abortus. Brucellosis* phages from environmental objects were isolated from a small amount of slurry taken from various locations of animal husbandry. To detect *Brucellosis* phages, 2-5 g of the slurry was thoroughly ground in sterile porcelain mortars; a small amount of the slurry was transferred into flasks that contained 50 cm^3^ of nutrient broth containing 1 billion microbial cells of a 2-day culture of *B. abortus* 19 vaccine strains. *Brucellae* culture containing inoculated biomaterial was subsequently grown in a thermostat at a temperature of 37°C for 3 days. One bacteriophage specific to the bacteria *B. abortus* was isolated as a result. The results indicated that the optimal temperature for the cultivation of bacterial phages is 35°C (Table-2). For higher accuracy, we decided to incubate the phage system with bacteria at a temperature of 35°C instead of the previously used temperature of 37°C.

**Table-2:** Temperature parameters of bacteriophage cultivation.

Bacteriophages	The temperature of phages and homologous bacteria cultivation			

	30°C	35°C	40°C	45°C
*Echerichiacoli* No. 1	+	+	+	−
*Proteus vulgaris* No. 13	+	+	+	−
*Proteus mirabilis* No. 45	−	+	−	−
*Yersinia pseudotuberculosis* No. 2	+	+	−	−
*Yersinia enterocolitica* No. 54	−	+	−	−
*Salmonella enteretidis* No. 15	−	+	−	−
*Salmonella typhimurium* No. 19	−	+	−	−
*Salmonella infantis* No. 21	−	+	−	−
*Enterococcus faecalis* No. 7	−	+	−	−
*Pseudomonas aeruginosa* No. 8	−	+	−	−
*Brucella abortus* No. 57	−	+	+	−
*Shigella sonnei* No. 61	−	+	−	−
*Shigella flexneri* No. 62	−	+	−	−

Note: “–“: No lysis, “+”: Lysis.

### Lytic activity of the isolated bacteriophages

**Figure-1:**
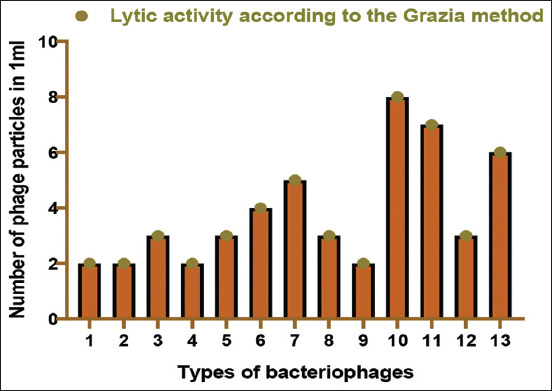
Lytic activity of bacteriophage titers according to Grazia.

Each culture of the studied bacteria was grown in a standard MIB for 18-20 h to determine the bacteriophage lytic activity. The lytic activity of the selected bacteriophages was determined using the Appelman and Grazia methods. In the Grazia method (determining the number of active phage particles in 1 mL of the substrate), the determination of the lytic activity of bacteriophages was performed by introducing the sample into 0.7% semiliquid agar containing a culture sensitive to the phage, followed by layering the mixture on dense 1.5% M-PA agar in a petri dish, incubating, and counting the number of negative colonies. Bacteriophages exhibited different lytic activity in bacterial cell cultures in a liquid medium and a solid medium. According to the Appelman and Grazia methods, the lytic activity of the isolated bacteriophages and the phage titers are presented in Table-3 and Figure-1, respectively. We found that polyphages cause lysis in the corresponding cultures. The lytic activity of phages is 10^–5^-10^9^ bodies in 1 cm^3^ according to the Appelman method and 2×10^8^–6 × 10^9^ bodies in 1 cm^3^ according to the Grazia method.

**Table-3:** Lytic activity of phages according to Appelman and phage titers according to Grazia.

Item No.	Type of bacteriophage active against bacteria	Lytic activity according to Appelman	Lytic activity according to Grazia. Number of phage particles in 1 mL
1	*Phagum Escherichia coli* No. 1	10^−8^	2×10^8^
2	*Phagum Proteus vulgaris* No. 13	10^−5^	8×10^8^
3	*Phagum Proteus mirabilis* No. 45	10^−5^	3×10^9^
4	*Phagum Yersinia pseudotuberculosis* No. 2	10^−7^	2×10^8^
5	*Phagum Yersinia enterocolitica* No. 54	10^−5^	4×10^9^
6	*Phagum Salmonella enteretidis* No. 15	10^−7^	4×10^9^
7	*Phagum Sallmonella typhimurium* No. 19	10^−7^	5×10^9^
8	*Phagum Sallmonella infantis* No. 21	10^−7^	3×10^8^
9	*Phagum Enterococcus faecalis*	10^−5^	2×10^9^
10	*Phagum Pseudomonas aeruginosa* No. 8	10^−7^	8×10^8^
11	*Phagum Brucella abortus* No. 57	10^−9^	7×10^8^
12	*Phagum Shigella sonnei* No. 61	10^−9^	3×10^8^
13	*Phagum Shigella flexneri* No. 62	10^−9^	6×10^9^

### Determination of virions of a group of bacteriophages by electron microscopy

**Figure-2:**
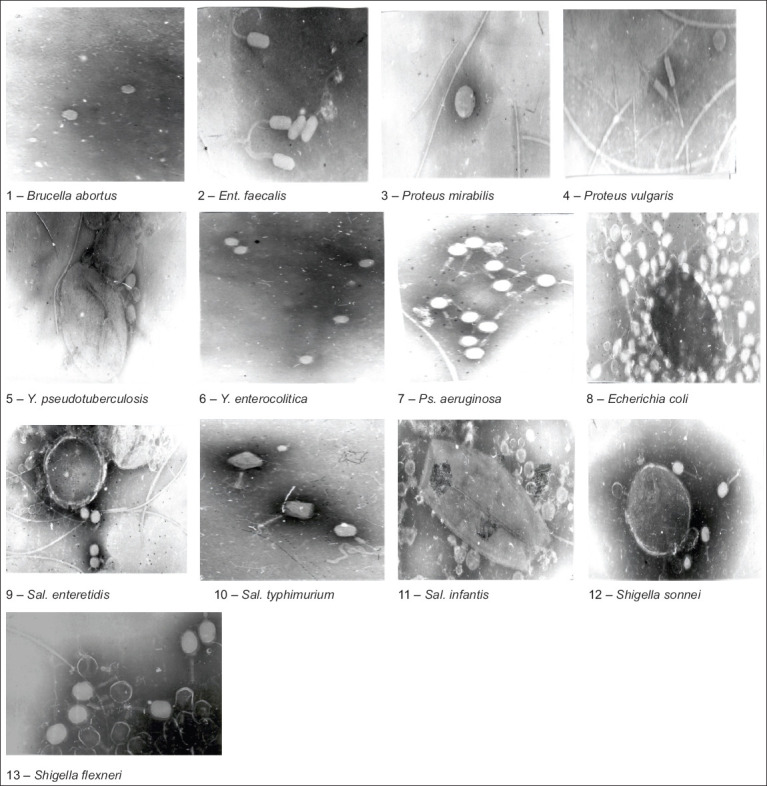
The results of the study by the method of electron microscopy of the polyphage/NIIPBB/BV-0001 consortium of bacteriophages. Distinguishable virions of bacteriophages were found in the field of view of the microscope.

For electron microscopy, bacteriophages under study were prepared by adsorption onto copper grids with a formvar substrate reinforced with carbon. Negative contrast was induced with a 2% aqueous solution of phosphoric–tungstic acid and examined on a JEM-100 CX-II JEOL transmission electron microscope (Japan) at an accelerating voltage of 80 kV and magnifications of 10,000×-20,000×. The study results are presented in Photos 1-10 (Figure-2). As a result of employing electron microscopy for the “Polyphage/NIIPBB/BV-0001” biological preparation consisting of a specific collection of bacteriophages, distinguishable virions of bacteriophages were found in the microscope field of view. These bacteriophages included spherical types with and without tail processes and were approximately 50-80 nm in size. We also observed ovoid phages with tail processes twice the diameter of the phage itself, hexagonal phages with tail processes, rod-shaped bacteriophages with and without caudal processes, and filamentous phages.

### Production of a pilot industrial sample of the “Polyphage” biological preparation with subsequent certification

Data obtained from the development of the polyphage biological preparation from a mixture of monophage phagolysates were used as the standard values of the cultivation parameters. The main parameters for the manufacture of monophagous phagolysates include the following: (1) The ratio of the bacteriophage to the culture sensitive to it; (2) the pH of the cultivation medium; and (3) the cultivation time of the bacteriophage system and the sensitive bacterium. The results indicate that the average ratio for all bacteriophages is 1:2, and the average pH value for all bacteriophages is 7.0. Finally, the average cultivation time for all bacteriophages is 18-24 h.

Cultural “mattresses” with a dense nutrient medium were inoculated on flasks for 18-24 h cultures of the aforementioned sensitive bacteria of each species of the consortium and then incubated in a thermostat for 6 h. After 6 h, each bacteriophage was inoculated on a nutrient-dense medium with sensitive bacteria in culture flasks. After 12 h of cultivation in a thermostat, they were washed with 30 mL of saline and treated with a 1:10 ratio of chloroform for 40 min using a vortex device to increase the speed of mixing of the contents of the test tubes through eccentricity and orbital rotation.

**Figure-3:**
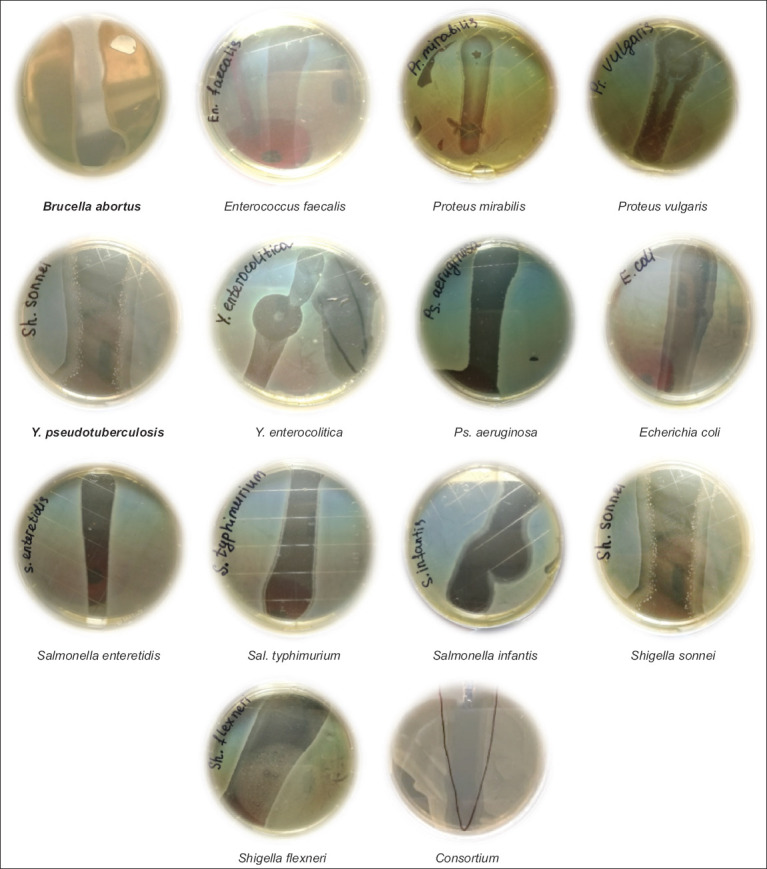
Activity of the used bacteriophages by the drop method.

Next, the phagolysate was centrifuged 2 times for 20 min at 3000rpm. The remaining 29 mL of supernatant was checked for the presence of viable bacterial cells. One mL of the supernatant was inoculated on nutrient agar in a Petri dish and cultured in a thermostat for 24 h. Another 1 mL was examined for phage activity using the Grazia method. The results obtained for each used bacteriophage and their corresponding consortiums are presented in Figure-3, which shows that all the studied phages lysed their corresponding bacteria.

### Tests to determine the shelf life of the “Polyphage” disinfectant

**Figure-4:**
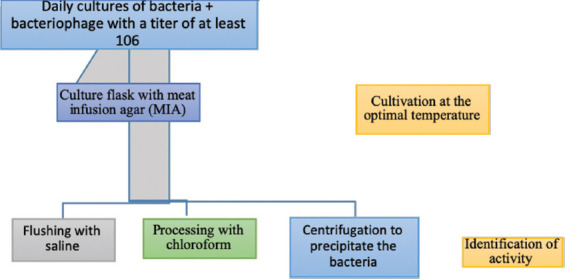
Diagram of the experimental production of the polyphage biological preparation.

The shelf life (i.e., stability) of the “Polyphage” preparation was determined using the Appelman method through titration on the GRM broth according to the following scheme: First, several test tubes containing 4.5 cm^3^ of the MIB medium were collected. “Polyphage” preparation (500 mL) was added into the first tube using a dispenser and mixed thoroughly with the other tip; 500 mL was transferred to the next tube. From the second tube, 500 mL was transferred to the third and so on. “Polyphage” was diluted in all series of test tubes in consecutively diminishing ratios of 1:10, 1:100, 1:1000, etc. A suspension of the culture was added to all test tubes, including the control tube, which contained only 4.5 cm^3^ of liquid medium. Subsequently, the tubes were placed and incubated, and the results were noted after the appropriate amount of time had elapsed. During phage titration, the phage and broth controls were set for sterility and control of the culture for its viability. Figure-4 presents a diagram of the pilot production of the polyphage biological preparations.

The results indicate that the greatest phage activity was observed in the pH of 6-8. The bacteriophages that are part of the biological preparation show resistance to chloroform at a concentration of 1-4%, allowing for the use of this reagent as a preservative and means for cleaning the phagolysate from bacterial cells. The preparation is non-pathogenic and non-toxic, which makes it possible for use in the field. Table-2 presents the results of the biological control of the biological preparation based on different phage groups.

By determining the cultivation parameters, the composition of phages in the biological preparation, and the indicator culture, the results indicate that the frequency of rotation of the stirrer neither significantly affect the bacterium growth rate nor the phage activity level. Cultivation at 200 rpm slightly slowed down the accumulation of bacterial biomass compared with cultivation at 160 rpm; this, in turn, increased the phase-induced lysis time of cells of the indicator culture.

Parameters of aeration levels were carried out at 0.5, 1.0, and 1.5l/l of medium per minute. The rotation speed of the mixer was set to 160 rpm, and the temperature was set to 28°C. The highest growth rate of the culture was observed at aeration levels of 1.0 and 1.5l/l of medium per minute. Furthermore, we found that the aeration level did not affect the lysis rate of bacterial cells. Approbation tests were conducted after identifying the parameters of the produced pilot sample of the “Polyphage” biological preparation.

The approbation tests consisted of the following tasks: (1) Testing to determine the external appearance (i.e., the integrity of the primary packaging, appearance, color, and smell); (2) determining purity, harmlessness, lytic activity, and shelf life; (3) electron microscopy of bacteriophage consortium “Polyphage/NIIPBB/BV-0001;” (4) control of the disinfection mode of the working equipment, disinfectant pans, and disinfectant barriers; and (5) production tests to determine the disinfection modes of the disinfectant “Polyphage/NIIPBB/BV-0001” in the slaughterhouse. The conclusion of tests revealed that the disinfectant “Polyphage/NIIPBB/BV-0001” corresponds to the parameters specified in the regulatory and technical documentation ST 405-1919-04 GP-105-2018.

## Discussion

Today, there is a global trend toward increasing bacterial resistance to antibacterial products. The World Health Organization (WHO) emphasizes that many of the drug treatment discoveries made in the 20^th^ century may become irrelevant due to antibiotic resistance. As a result, many infectious diseases may become unmanageable. While currently used antibacterial preparations rapidly lose their effectiveness, new products are slowly developing. If this trend continues, the arsenal of tools to fight resistant microorganisms may become exhausted. Along with the growing resistance of most infectious pathogens, the development of antibiotic resistance in pathogens is reaching alarming proportions. Antimicrobial resistance has developed in leading pathogens. Some examples include methicillin-resistant *Staphylococcus aureus*, *E. coli*, and *Klebsiella pneumoniae* (which produce beta-lactamase of the wide and extended spectrum); *P. aeruginosa* and *Acinetobacter baumannii* are resistant to carbapenems. Vancomycin-resistant enterococcus pathogens *Enterococcus faecium*, *Enterococcus faecalis*, and several other microorganisms are among this list. As antibiotic resistance increases and the rate of development of new antibacterial agents declines, the WHO and leading public health organizations in many countries point to the urgent need to address the problem of drug resistance, calling for full support for efforts to introduce new approaches to fight bacterial infections [4,24-26].

The range of treatment and preventive measures for infectious diseases is gradually decreasing due to the global increase in antimicrobial resistance of microorganisms. Among the primary tasks are the development and application of additional means for fighting bacterial diseases, in which bacteriophages show potential. The use of bacteriophages, like any antibacterial preparations, should be based on rational principles. Moderate bacteriophages play a significant role in bacterial evolution, promoting the acquisition of additional virulence factors by pathogens. Bacteriophages used to treat and prevent bacterial diseases should be exclusively virulent. To ensure this, the lytic activity of bacteriophage preparations prescribed for treatment should be pre-tested in a bacteriological laboratory [27,28].

In the foreign market, the United States has the leading position in the research on products containing bacteriophages. However, these products are undergoing clinical trials and approvals with the competent supervisory authorities and hence have not yet been presented on the market. Bacteriophages have been widely used to treat various diseases since the 1920s, both in the United Socialist Soviet Republic and in Western countries. However, since the 1950s, the production and use of phages in Western countries have ceased. Bacteriophage preparations are currently produced in Russia, Georgia, and Poland. The latest data indicate a renewed interest in phage therapy, as a result some efforts are being made to revive the practice of using bacteriophage preparations [11,12].

### Analysis of the current market for disinfectants of close equivalents of “Bacteriophage”

The latest report, “bacteriophage market – Growth, Future, Competitive Analysis, 2018-2026,” published by the American company Credence Research, estimated the global bacteriophage market of USD 567 million in 2017 and forecasts an average annual growth rate of 3.9%. The report states that bacteriophages (i.e., phages) are now seen as an important tool in modern biotechnology, as well as an alternative to antibiotics in the treatment of antibiotic-resistant infections. Furthermore, phages can be used for biological control in agriculture and power industries, particularly, to control the bacterial corrosion of gas pipelines. Bacteriophages can be used as delivery agents for vaccines, for the detection of pathogenic bacteria in various environments, and as the basis of biosensors and phage display technology. Phages can destroy bacteria and transfer genetic information between them; hence, their study will help understand the mechanisms of genome evolution, bacterial evolution, and DNA expression and, consequently, will serve to create new biotechnologies. The global increase in the number of pathogenic bacteria resistant to antibiotics has stimulated the use of phages in medicine. In clinical use, specific phages do not affect eukaryotic cells or destroy normal human microflora. An important sphere of the application of phages is the food industry, where phage preparations are used for the antimicrobial treatment of food products and equipment. The bacteriophage market is divided into the following segments: Phages with single-stranded DNA, double-stranded DNA (dsDNA), and single-stranded RNA. In 2017, dsDNA phages were the leading segments; they are expected to continue dominating the market. Geographically, most of the bacteriophage market is concentrated in the USA due to the abundance of phage research and manufacturing companies, the fastest product commercialization process, and a large number of patients with antibiotic-resistant infections. The report also notes the dynamic development of the bacteriophage market in the Asia-Pacific region, mainly due to government initiatives [29].

On the Kazakh website “Tender+,” antimicrobial products and disinfectants are based on chemicals: Glutaraldehyde, sodium percarbonate, organochlorine compounds, alcohol, ammonium, aldehydes, and quaternary ammonium compounds, many of which can cause side effects, such as allergies and oncological diseases; our “Polyphage” product has no such drawbacks. The following manufacturers are also presented: “World of Disinfection, LLC” (Russia) has a disinfectant named “Miroseptik” that costs KZT 3440/L, and “Scientific-Production Association MediDez, LLP” (Kazakhstan) has the preparation “Farmdezin Ultra” that costs KZT 6348/L. In addition, “NPO MediDez, LLP” has the preparation “Medidezenzo” (a concentrate), a product based on five enzymes, which cost KZT 21,800/L [30]. After analyzing data from the website “Tender+” and results of telephone surveys of the veterinary pharmacy networks “Bios,” “Vita,” “GeoSintez,” and “Kazvetsnab,” we concluded that there are no bacteriophage-based preparations among the presented antimicrobial and disinfectants. The closest analog, “Medidezenzo,” is sold at a high price of KZT 21,800/L. Thus, the KZT 3000/L price of the disinfectant product “Polyphage” is reasonable and is the most profitable competitive offer [30]. Kazakhstan has a huge opportunity to commercialize bacteriophages and capture a wider spectrum of the world market due to its participation in the Tripartite Alliance and the multicountry EurAsEC Customs Union [31].

Veterinary science undergoes a constant search for the best strategies and tactics to maintain the health of productive animals, ensure food security, and obtain environmentally friendly, high-quality livestock products. The need for antimicrobials that are effective against modern pathogens and do not inhibit normal flora has led researchers to turn their attention to bacteriophages, which have been successfully used for treating intestinal infections even before the discovery of antibiotics. The therapeutic and preventive bacteriophages contain highly virulent bacteriophages of a highly specific nature. The main advantages of bacteriophage preparations include the high sensitivity of pathogenic microflora to bacteriophages, their ability to combine with all types of conventional antibacterial therapies, and the lack of contraindications of phage prophylaxis and phage therapy. Given the polyetiology of many diseases caused by opportunistic pathogens, the development of polyvalent combined bacteriophages is promising [3,32].

The proposed “Polyphage” biological preparation selectively inactivates a wide spectrum of bacterial species, such as *B. abortus*, *E. faecalis*, *P. mirabilis*, *P. vulgaris*, *Y. pseudotuberculosis*, *Y. enterocolitica*, *P. aeruginosa*, *E. coli*, *S. enteritidis*, *S. typhimurium*, *S. infantis*, *Shigella sonnei*, and *S. flexneri*, in the said premises for other purposes. Furthermore, the addition of the preservative catamin AB helps protect equipment against corrosion without affecting the bacteriophages.

There are several advantages of the “Polyphage” biological preparation, developed during the implementation of the project, over existing commercial preparations. The titers of bacteriophages in the biological preparation have at least 10^5^ active particles compared to the 10^3^ of existing medicinal products. The “Polyphage” biological preparation differs from the traditional means of preventing bacterial infections in that it has a prolonged effect due to the self-reproduction of bacteriophages; it is hypoallergenic, non-toxic, non-flammable, and explosion proof. Finally, it does not violate the natural balance of microflora.

The production of a biological preparation assumes a clear understanding and reproduction of complex chemical and biological processes involved in the interaction of a bacteriophage with a bacterial cell; strict adherence to technological regimes is necessary. The technological aspect of the project involves the development of critical parameters for the manufacture of the new “Polyphage” biological preparation as the basis for its industrial production for a wide range of consumer applications.

## Conclusion

As a result of the phage typing assays conducted, 11 bacteriophages were isolated; one bacteriophage was specific to *P. aeruginosa*, and one was specific to *B. abortus*. All strains of bacteriophages demonstrated lysis at a pH of 7.0. The study results indicate that the optimum temperature for bacterial phage cultivation is 35°C. In addition, the polyphages lyse all strains of sensitive bacterial cultures. The results indicate that the level of aeration does not affect the lysis rate of bacterial cells. Bacteriophages, which are part of the biological preparation, exhibit resistance to chloroform at a concentration of 1-4%, making it possible to use this reagent as a preservative and a means for purifying phagolysate from bacterial cells. Electron microscopy of a consortium of bacteriophages in the microscope field of view showed the presence of clearly distinguishable virions of bacteriophages.

## Authors’ Contributions

SSI: Analysis and interpretation of data. NSS: Drafted the manuscript. AAS: Conception and design of the study. KDA: Revised the manuscript critically. BAY: Data collection and analysis. All authors read and approved the final manuscript.
